# ROS-Mediated Necroptosis Is Involved in Iron Overload-Induced Osteoblastic Cell Death

**DOI:** 10.1155/2020/1295382

**Published:** 2020-10-16

**Authors:** Qing Tian, Bo Qin, Yufan Gu, Lijun Zhou, Songfeng Chen, Song Zhang, Shuhao Zhang, Qicai Han, Yong Liu, Xuejian Wu

**Affiliations:** ^1^Department of Orthopaedics, The First Affiliated Hospital of Zhengzhou University, Zhengzhou 450052, China; ^2^Department of Translational Medical Center, First Affiliated Hospital of Zhengzhou University, Zhengzhou 450052, China; ^3^Department of Orthopaedics, Union Hospital, Tongji Medical College, Huazhong University of Science and Technology, Wuhan 430022, China

## Abstract

Excess iron has been reported to lead to osteoblastic cell damage, which is a crucial pathogenesis of iron overload-related osteoporosis. However, the cytotoxic mechanisms have not been fully documented. In the present study, we focused on whether necroptosis contributes to iron overload-induced osteoblastic cell death and related underlying mechanisms. Here, we showed that the cytotoxicity of iron overload in osteoblastic cells was mainly due to necrosis, as evidenced by the Hoechst 33258/PI staining, Annexin-V/PI staining, and transmission electronic microscopy. Furthermore, we revealed that iron overload-induced osteoblastic necrosis might be mediated via the RIPK1/RIPK3/MLKL necroptotic pathway. In addition, we also found that iron overload was able to trigger mitochondrial permeability transition pore (mPTP) opening, which is a critical downstream event in the execution of necroptosis. The key finding of our experiment was that iron overload-induced necroptotic cell death might depend on reactive oxygen species (ROS) generation, as N-acetylcysteine effectively rescued mPTP opening and necroptotic cell death. ROS induced by iron overload promote necroptosis via a positive feedback mechanism, as on the one hand N-acetylcysteine attenuates the upregulation of RIPK1 and RIPK3 and phosphorylation of RIPK1, RIPK3, and MLKL and on the other hand Nec-1, siRIPK1, or siRIPK3 reduced ROS generation. In summary, iron overload induced necroptosis of osteoblastic cells in vitro, which is mediated, at least in part, through the RIPK1/RIPK3/MLKL pathway. We also highlight the critical role of ROS in the regulation of iron overload-induced necroptosis in osteoblastic cells.

## 1. Introduction

Iron, an essential micronutrient, plays a crucial role in a wide scale of biological processes like DNA synthesis, energy metabolism, and oxygen transport; however, excess iron is toxic to cells as leading to organ dysfunction and diseases [1, 2]. As reported, excess iron stored in the bone tissue is linked with higher rates of bone loss at proximal femur sites even in healthy people [3]. Moreover, patients with iron overload associated diseases like hemochromatosis, thalassemia, and sickle cell disease are much more prone to suffer from osteoporosis [4, 5]. Nevertheless, the fundamental mechanisms by which iron overload causes osteoporosis remain poorly understood.

Recently, substantial evidence has accumulated to demonstrate that oxidative stress caused by iron overload is the major contributor to the pathogenesis of osteoporosis [6–8]. In our previous study, we have demonstrated that reactive oxygen species was essential for iron overload-induced apoptosis in the osteoblastic cells [9]. More importantly, an in vivo study of iron overload documented that elimination of ROS by antioxidants largely prevented the bone abnormalities and inhibited detrimental inflammatory cytokine production [10, 11]. However, it has been documented that apoptosis is generally considered to be nonimmunogenic [11, 12]. Meanwhile, we also noticed that osteoblastic cell death was only partially mediated by apoptosis under iron overload conditions [9]. Based on previous related studies and this phenomenon, we hypothesized that other types of cell death might account for the underlying mechanisms.

Necroptosis is another type of programmed cell death characterized by cellular organelle swelling and membrane rupture, induced by multiple death receptors, oxidative stress, or mitochondrial dysfunction, which is mainly mediated by the RIPK1/RIPK3/MLKL pathway [13–15]. Recent studies have suggested that necroptosis plays an important role in the regulation of tissue homeostasis and disorders [16–18]. It has also been established that activation by stimuli leads to RIPK1 autophosphorylation, recruitment of RIPK3 to RIPK1 to form the necrosome complex, then oligomerization of MLKL, and finally translocation to the plasma membrane to execute necroptotic cell death [19]. However, it is unknown whether necroptosis was implicated in iron overload-induced osteoblastic cell death.

In our current study, for the first time, we systematically confirm that the necroptosis pathway is involved in iron overload-induced death of osteoblastic cells. The key finding of this study is that ROS is essential for iron overload-induced necroptosis. We then further discover that ROS contribute to necroptosis of osteoblastic cells through a positive feedback loop involving RIPK1/RIPK3. These findings suggest targeted antioxidants as an alternative therapy for the prevention and treatment of iron overload relevant osteoblastic cell injury.

## 2. Materials and Methods

### 2.1. Cell Culture

MC3T3-E1 (subclone 4; ATCC® CRL-2593™), an osteoblast cell line, was obtained from American Type Culture Collection [20, 21]. Cells were maintained in modified *α*-Minimum Essential Medium Eagle (*α*-MEM) (HyClone, Logan, USA) which contains 10% fetal bovine serum (FBS) (Gibco, Invitrogen, NY, USA) and 1% antibiotics (penicillin and streptomycin) in a humidified atmosphere at 37°C. The culture medium was replaced every 2 or 3 days.

### 2.2. Treatment Groups

Ferric ammonium citrate (FAC) (Sigma, St. Louis, MO, USA), a source of iron, was utilized to mimic iron overload conditions in vivo and in vitro [7, 9, 22]. To evaluate the cytotoxic effect of iron overload, the MC3T3-E1 osteoblastic cells were exposed to FAC (50, 100, and 200 *μ*M) for 24, 72, and 120 h, respectively. The control groups were treated with 0.9% saline solution. According to our previous experiments, the cell viability of osteoblastic cells significantly decreased from 24 to 120 h [9]. More importantly, with iron overload exposure time prolonged, the osteoblastic cell necrosis peaked at 120 h. Thus, 120 h FAC-treated time periods were chosen throughout the following study. N-acetyl-cysteine (NAC) (Beyotime Biological Technology, Shanghai, China) was dissolved in phosphate-buffered saline (PBS). The RIPK1 inhibitor Necrostatin-1 (Nec-1) (Selleck, Houston, TX), MLKL inhibitor Necrosulfonamide (NSA) (Selleck, Houston, TX), and RIPK3 inhibitor GSK872 (Selleck, Houston, TX) were dissolved in DMSO solution. Before exposure to FAC, the MC3T3-E1 osteoblastic cells were incubated with or without NAC (1 mM), Nec-1 (20 *μ*M), GSK872 (4 *μ*M), or NSA (4 *μ*M) [9, 23–25]. Then, after FAC (200 *μ*M) treatment for 120 h, all samples were collected and analyzed by a microplate reader, flow cytometry, western blots, and confocal microscopy.

### 2.3. Cell Counting Kit-8 Assay

The cytotoxicity of iron on osteoblastic cells was determined by the cell counting kit-8 assay kit (Dojindo Biotechnology, Japan) as described in instruments [9]. The osteoblastic cells were collected and seeded in a 96-well plate. After culture for 24 h, the osteoblastic cells were treated with FAC as described above. Then, the culture medium was removed and replaced with 100 *μ*l mixture solution containing 10 *μ*l of CCK-8 reactant. After reaction for 2 h at room temperature in the dark, the absorbance value at 450 nm was detected in a microplate reader (Thermo, Waltham, MA, USA).

### 2.4. Evaluation of Cell Death by Annexin-V-FITC/Propidium Iodide Staining

After exposure as mentioned above, the osteoblastic cells were stained using Annexin-V-FITC/propidium iodide (PI) kit (KeyGen Biotech, Nanjing, China) [9]. Then, the osteoblastic cells underwent trypsinization and centrifugation. Subsequently, cells were gently washed twice by PBS and resuspended in 500 *μ*l binding buffer containing 5 *μ*l Annexin-V and 5 *μ*l PI. After incubating for 15 min in the dark, the samples were detected using flow cytometry (Becton Dickinson, NJ, USA).

### 2.5. Hoechst 33258/Propidium Iodide Staining

Cell death was evaluated by the confocal laser scanning microscope (OLYMPUS FV1000, Japan) using Hoechst 33258/PI staining kit (Sigma, St. Louis, MO, USA) [26]. The osteoblastic cells were seeded and treated in 24-well culture plates as described above. Then, the culture medium was discarded, and the plates were washed twice with PBS. Hoechst 33258 (10 mg/ml) and PI (5 mg/ml) were used to stain osteoblastic cells. After incubating for 10 min in the dark, the osteoblastic cells were washed with phosphate-buffered saline and observed under the confocal laser scanning microscope.

### 2.6. Transmission Electron Microscopy (TEM)

The cellular ultrastructure of osteoblasts after treatment with FAC was observed by TEM as previously described [23]. Briefly, the osteoblastic cells were collected, centrifuged, and rinsed twice with PBS. Next, all samples were fixed in 2.5% glutaraldehyde for 2 h and subsequently postfixed with 1% osmium tetroxide for 2 h at 37°C. Then, after being dehydrated with ethanol and embedded in Epon-812, the ultrathin sections were stained with lead citrate and uranyl acetate and were used to evaluate ultrastructure changes with TEM (Tecnai 20 200 kV, FEI Company, Holland).

### 2.7. Western Blot Analysis

When the cells reached an 80-90% confluence, they were then seeded at 6 × 10^4^ cells/cm^2^ density onto appropriate culture plates for western blot. After treatment as described above, the osteoblastic cells were harvested and lysed in the RIPA lysis buffer (Boster Biological Technology, Wuhan, China). After centrifugation at 12, 000∗g for 10 min at 4°C, the protein concentrations were determined by an Enhanced BCA Protein Assay Kit (Beyotime Biological Technology, Shanghai, China). Then, the total lysates of each simple were separated by 12% SDS-PAGE and then transferred to the nitrocellulose membranes, which was blocked in blocking solution for 2 h at room temperature and incubated with primary antibodies overnight at 4°C [27]. All the primary antibodies used in this experiment were the following: anti-RIPK1 (1 : 500, CST, USA), anti-Phospho-RIPK1 (1 : 1000, CST, USA), anti-RIPK3 (1 : 1000, Abcam, USA), anti-Phospho-RIPK3 (1 : 1000, Abcam, USA), anti-MLKL (1 : 1000, CST, USA), anti-Phospho-MLKL (1 : 1000, CST, USA), anti-VDAC1 (1 : 1000, CST, USA), anti-PGAM5 (1 : 1000, Abcam, USA), anti-Drp-1 (1 : 1000, Abcam, USA), anti-Phospho-Drp-1 (ser637) (1 : 1000, Abcam, USA), and anti-*β*-Actin (1 : 1000, Abcam, USA). Following three washes by TBST solution, membranes were incubated with horseradish peroxidase-conjugated secondary antibodies for 2 h at room temperature. Finally, the protein levels were determined by the enhanced chemiluminescence kit (Boster Biological Technology, Wuhan, China) as described in the manufacturer's instructions.

### 2.8. Estimation of Mitochondrial Membrane Potential (MMP)

MMP was quantified by flow cytometry after stained in 5,5′,6,6′-tetrachloro-1,1′,3,3′-tetraethyl-benzimidazolycarbocyanine iodide (JC-1) (Beyotime Biological Technology, Shanghai, China). When the mitochondrial membrane potential is high, JC-1 aggregates in the matrix which can produce red fluorescence. When MMP is low, JC-1 disperses which can exhibit green fluorescence. In this study, the osteoblastic cells were subjected as described above and stained according to the manufacturer's instructions [28]. The levels of MMP in osteoblastic cells were analyzed by flow cytometry and determined by the ratio of red to green fluorescence intensity. Additionally, a laser scanning confocal microscopy (OLYMPUS FV1000, Japan) was utilized to visualize the changes of MMP in situ.

### 2.9. Evaluation of Mitochondrial Permeability Transition Pore (mPTP)

The mPTP of the osteoblastic cells was analyzed by mPTP Assay Kit (Beyotime Biological Technology, Shanghai, China) as previously described [24]. Finally, the relative fluorescence intensity (RFI) in different groups was determined by flow cytometry.

### 2.10. Detection of Reactive Oxygen Species

2',7'-Dichlorofluorescin diacetate (H2DCF-DA) (Sigma, St. Louis, MO, USA) is a special and commonly used fluorescent probe to detect the intracellular ROS level. Briefly, after being treated as described above, the culture medium was discarded and the osteoblastic cells were harvested and washed thrice by PBS. Then, cells were resuspended and incubated with 20 *μ*M H2DCF-DA at 37°C for 20 min in the dark [25]. Subsequently, serum-free medium was used to rinse the cells in order to remove the residual dyes. Finally, the mean fluorescence intensity (MFI) in different groups was determined by flow cytometry. In addition, in order to estimate the production of intracellular ROS levels in situ, a fluorescence microscope (Carl Zeiss, German) was utilized to visualize each sample.

### 2.11. siRNA Knockdown

siRIPK1 and siRIPK3 were synthesized by Ribo Biological Technology (Ribo, Guangzhou, China) according to the current guidelines [23, 24, 29, 30]. The sequences of each are shown in Table 1.

Nonspecific siRNA was treated as a negative control group (siControl).

The osteoblastic cells were transfected with effective sequence at a concentration of 100 pmol/10^5^ cell using Lipofectamine™ RNAi MAX reagent (Invitrogen, Life Technologies) according to the manufacturer's protocol [23, 24]. After 24 h transfection with siRNAs, the osteoblastic cells were used for subsequent experiments. The knockdown efficiency of the siRNAs was assessed by western blotting.

### 2.12. Statistical Analysis

All data were derived from at least three separate experiments and were given as the mean ± standard deviation (SD). Statistical analyses were performed with SPSS software package 18.0 by the method one-way analysis of variance (ANOVA) to compare different samples. Student's *t*-tests were also performed to analyze the differences between the two groups. A probability of *p* < 0.05 was considered statistically significant.

## 3. Results

### 3.1. Iron Overload Impair the Viability of Osteoblastic Cells

**Figure 1:**
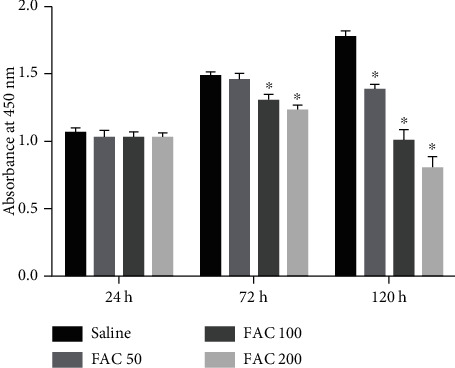
The influence of iron overload on the osteoblastic cell viability. After subjecting to FAC (50-200 *μ*M) for 24, 72, and 120 h, the viability of osteoblasts was detected by CCK-8 assays. The histogram showed the statistical significance only in the 72 h and 120 h groups. Values are expressed as the means ± SD from three independent experiments (^∗^*p* < 0.05 vs. saline control, ANOVA test).

To explore the cytotoxic effects of iron overload on the osteoblastic cells, CCK-8 assays were used to quantitate cell viability. The results showed the dose-dependent cytotoxicity as evidenced by the decrease of absorbance in the osteoblastic cells with increasing FAC treatment for 72 h and 120 h (Figure 1). However, there is no statistical difference between control groups and treatment groups for 24 h, suggesting iron exerts cytotoxic effects through a long-term accumulation in the osteoblastic cells. Thus, the osteoblastic cells were subjected to FAC (50-200 *μ*M) for 120 h in the following experiments.

### 3.2. Characterization of Osteoblastic Cell Death Induced by Iron Overload

**Figure 2:**
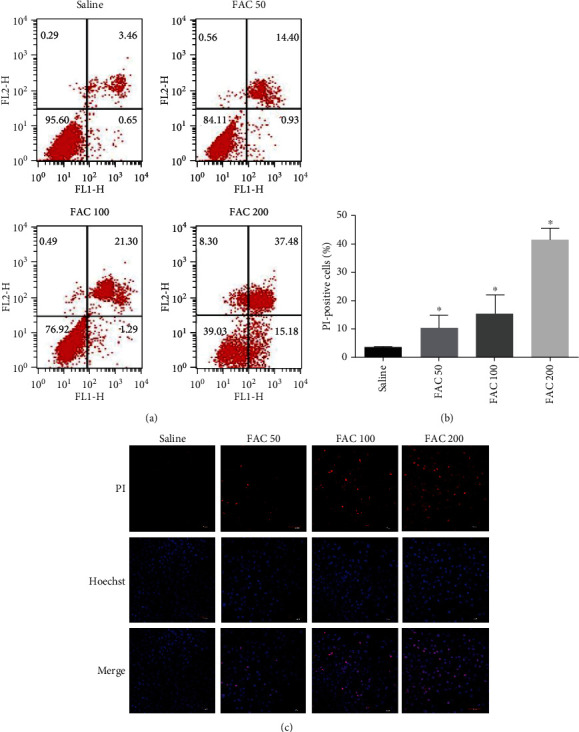
Iron overload-induced necrotic cell death in the osteoblastic cells. (a) Representative data were analyzed using the flow cytometer after Annexin-V/PI staining in osteoblastic cells after exposure to 50-200 *μ*M FAC for 120 h. In each plot, the lower left quadrant (Annexin-V-/PI-) corresponds to live cells, the lower right quadrant (Annexin-V+/PI-) corresponds to apoptosis, and the upper left and right quadrants (Annexin-V-/PI+ and Annexin-V+/PI+) correspond to necrotic cell. (b) Histogram statistical analysis illustrated the dose-dependent increase of PI-positive osteoblasts. Values are expressed as the means ± SD from three independent experiments (^∗^*p* < 0.05 vs. saline control, ANOVA test). (c) Representative confocal photomicrograph of Hoechst 33258/PI staining. After treatment with FAC (50-200 *μ*M) for 120 h, the osteoblastic cells were counterstained with Hoechst 33258 and PI. PI positive represents necrotic cells. Scale bar = 30 *μ*m.

**Figure 3:**
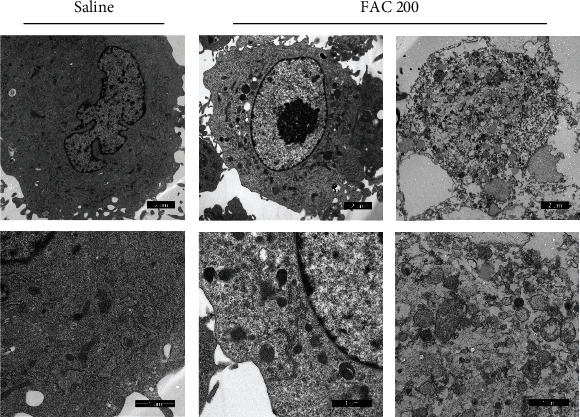
Typical TEM images of morphological ultrastructural changes in the osteoblastic cells. In the saline control group, the osteoblastic cells exhibited a normal cell morphology as manifested by intact plasma membrane and organelles. After exposure to 200 *μ*M FAC for 120 h, the osteoblastic cells displayed typical necrotic ultrastructural changes including losing integrity of the plasma membrane, swelling of organelles, and eventually cellular lysis. Scale bar = 1 *μ*m and 2 *μ*m.

In order to analyze the type of osteoblastic cell death induced by iron overload, Annexin-V/PI staining and Hoechst 33258/PI staining were used to detect cell death. In our study, after incubating with FAC (50-200 *μ*M) for 120 h, the increase of osteoblastic cell death was mainly caused by the PI-positive cells (Figures 2(a)). Meanwhile, as shown in Figures 2(b), there is a definitive increase in the ratio of PI-positive cells with elevated dose of FAC (*p* < 0.05). Consistently, the Hoechst 33258/PI staining demonstrated that the ratio of necrosis (PI-positive cells) was elevated greatly in osteoblastic cells after treatment with FAC (50-200 *μ*M) (Figures 2(c)). To further identify that cellular necrosis was induced by iron overload in the osteoblastic cells, TEM was applied to observe morphological changes. With the detection by TEM, after treatment with 200 *μ*M FAC for 120 h, the osteoblastic cells presented typical necrotic morphological features including severe vacuolation, organelle swelling, and subsequent cellular lysis (Figure 3). Taken together, our results of experiments demonstrated that iron overload-induced osteoblastic cell death might be mainly due to cellular necrosis.

### 3.3. Implication of the Necroptotic Pathway in Iron-Induced Osteoblastic Necrosis

**Figure 4:**
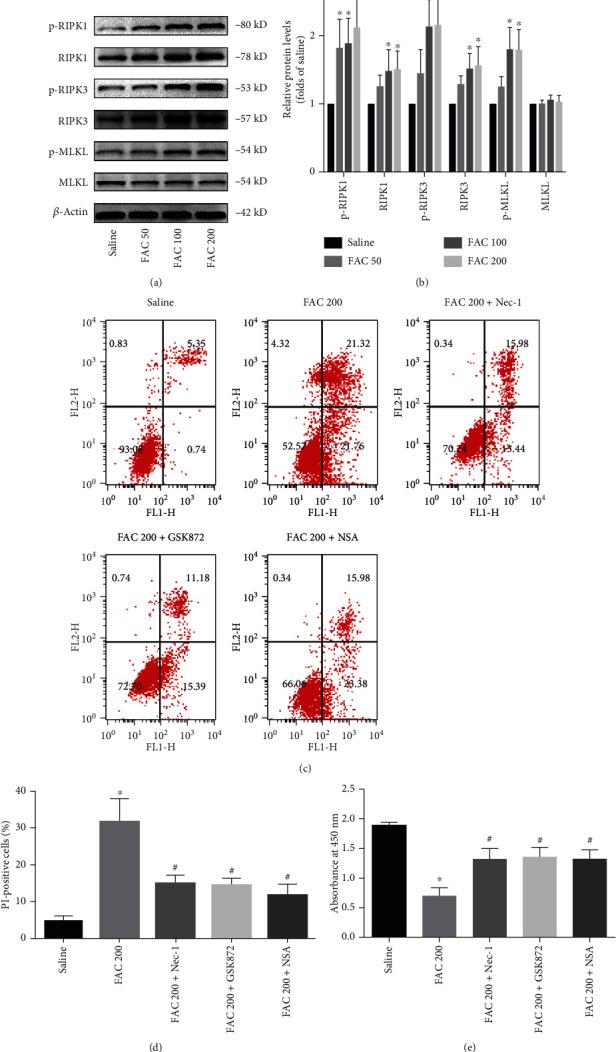
Implication of the necroptosis pathway in iron-induced osteoblastic necrosis. (a) Total protein levels of phosphorylated RIPK1, RIPK3, MLKL, and corresponding total protein. After exposure to FAC (50-200 *μ*M) for 120 h, western blot was used to detect the expression of phosphorylated RIPK1, RIPK3, MLKL, and corresponding total protein in osteoblastic cells. (b) Histogram analysis showing the relative protein levels of phosphorylated RIPK1, RIPK3, MLKL, and corresponding total protein. Data are presented as the means ± SD from three independent experiments (^∗^*p* < 0.05 vs. saline control, ANOVA test). (c) Representative data were analyzed using the flow cytometer after Annexin-V/PI staining in osteoblastic cells after exposure to 50-200 *μ*M FAC for 120 h. (d) Histogram statistical analysis demonstrating the ratio of PI positive cells. Values are expressed as the means ± SD from three independent experiments (^∗^*p* < 0.05 vs. saline control, ^#^*p* < 0.05 vs. FAC 200, ANOVA test). (e) The protective effects of Nec-1, GSK872, or NSA on the osteoblastic cell viability were evaluated by the CCK-8 assay. Values are expressed as the means ± SD from three independent experiments (^∗^*p* < 0.05 vs. saline control, ^#^*p* < 0.05 vs. FAC 200, ANOVA test).

Necroptosis, one form of programmed cellular necrosis, recently has been demonstrated to play an essential role in several pathologies [11]. To explore whether iron-induced osteoblastic cell death is associated with necroptosis, we detected the expression of RIPK1, RIPK3, and MLKL, three crucial regulating molecules of the necroptosis pathway. As shown in Figures 4(a) and 4(b), after exposure to FAC (50-200 *μ*M) for 120 h, the expression levels of RIPK1 and RIPK3 were upregulated in a dose-dependent manner. However, FAC had no significant modulatory effect on the expression of MLKL. Interestingly, the phosphorylation of RIPK1, RIPK3, and MLKL in the osteoblastic cells was increased in response to FAC treatment. To further confirm whether the RIPK1-RIPK3-MLKL pathway was involved in the necroptosis induced by iron overload, the osteoblastic cells were pretreated with and without the Nec-1 (20 *μ*M) (Supplementary Figure 1), GSK872 (4 *μ*M) (Supplementary Figure 2), and NSA (4 *μ*M) (Supplementary Figure 3), respectively. As shown in Figures 4(c) and 4(d), the ratio of PI-positive cells was effectively decreased by Nec-1, GSK872, or NSA. Meanwhile, the data of CCK-8 assays demonstrated that the osteoblastic cell viability was effectively improved by GSK872, Nec-1, or NSA (Figure 4(e)). Taken together, these findings illustrate that RIPK1, RIPK3, and MLKL are required for iron overload-induced necrotic cell death in osteoblastic cells.

### 3.4. Iron Overload Induces the Opening of Mitochondrial Permeability Transition Pore (mPTP) in Osteoblastic Cells

**Figure 5:**
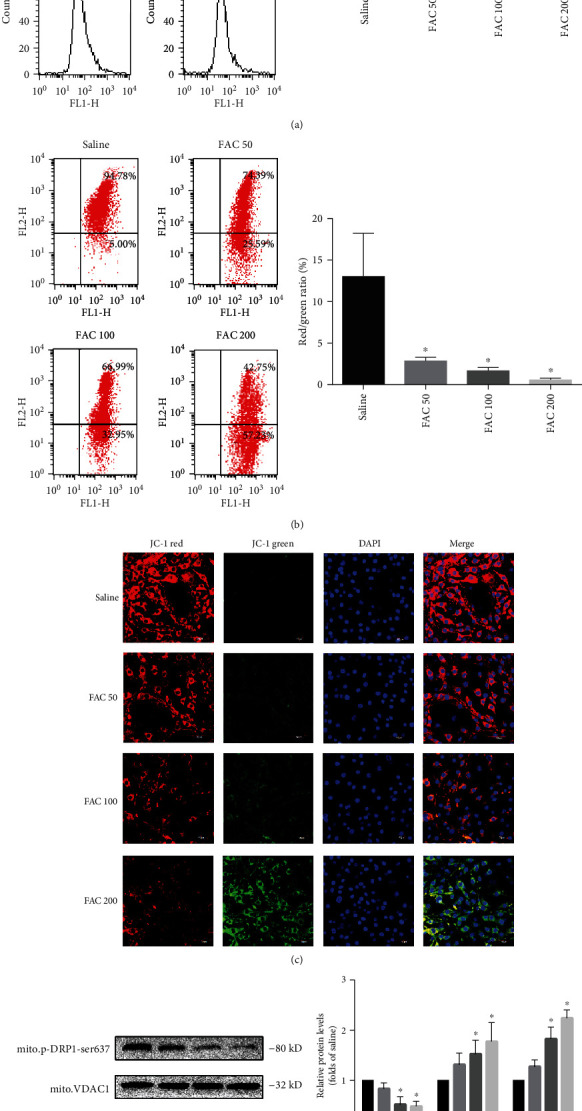
Iron overload induces the opening of mitochondrial permeability transition pore in osteoblastic cells. The opening of mitochondrial permeability transition pore results in the loss of mitochondrial membrane potential. After exposure to FAC (50-200 *μ*M) for 120 h, the flow cytometry was used to detect the opening of mPTP and changes of MMP. (a) Representative dot plot of the flow cytometry results illustrated the increase of mPTP opening. The quantitative mPTP in osteoblasts is shown by RFI. Values are expressed as the means ± SD from three independent experiments (^∗^*p* < 0.05 vs. saline control, ANOVA test). (b) Representative dot plot of the flow cytometry results illustrated the decrease MMP. The quantitative MMP in osteoblasts is shown by red/green ratio. Values are expressed as the means ± SD from three independent experiments (^∗^*p* < 0.05 vs. saline control, ANOVA test). (c) Representative fluorescence images were obtained in situ JC-1 staining by confocal microscopy. Scale bar = 50 *μ*m. (d) Representative western blots of the total protein expression of PGAM5, DRP1, and mitochondrial protein expression of phosphorylated DRP1 (Ser637). (e) Histogram analysis showing the relative protein levels of PGAM5, DRP1, and mitochondrial protein expression of phosphorylated DRP1 (Ser637). Data are presented as the means ± SD from three independent experiments (^∗^*p* < 0.05 vs. saline control, ANOVA test).

Mitochondrial dysfunctions have been considered a crucial step during iron overload-induced cell death [31]. Various pathologic factors could trigger the opening of mitochondrial permeability transition pore, result in the loss of mitochondrial membrane potential, and eventually lead to necroptosis. To illustrate the fundamental mechanisms of iron overload-caused necroptosis, we explored its possible effects on mitochondria. Firstly, we detected the opening of mPTP by flow cytometry in osteoblastic cells. As shown in Figure 5(a), the value of RFI was markedly decreased in osteoblastic cells after treatment with FAC (50-200 *μ*M) for 120 h. Meanwhile, we also estimated the changes of mitochondrial membrane potential by JC-1 staining. The collapse of the mitochondrial membrane potential was defined as the decline in the ratio of red fluorescence (JC-1 aggregates) to green fluorescence (JC-1 monomers). After exposure to FAC (50-200 *μ*M) for 120 h, compared with the saline group, a dose-dependent decrease of the ratio of red to green fluorescence in osteoblastic cells was detected by flow cytometry (Figure 5(b)). To further evaluate the loss of mitochondrial membrane potential caused by iron overload, after JC-1 staining, the osteoblastic cells were detected by the confocal microscopy in situ. In the saline group, the osteoblastic cells exhibited primarily red fluorescence, indicating that the mitochondrial membrane potential was normal. However, after treatment with FAC (50-200 *μ*M) for 120 h, the red fluorescence in the osteoblastic cells was dramatically decreased and the green fluorescence was increased (Figure 5(c)).

Next, to further explore the molecular pathway underlying iron overload-induced mitochondrial permeability transition pore opening, we determined the alteration of PGAM5 and DRP1, two important molecules in maintaining mitochondrial homeostasis, which have been uncovered to act as a substrate of RIPK3. PGAM5, once activated by RIPK3, will be recruited to mitochondrial membrane, which then actives DRP1 by dephosphorylation of serine 637 site and ultimately results in mPTP opening and necroptosis [32]. As shown in Figures 5(d) and 5(e), following 50-200 *μ*M treatment, the total protein expression of PGAM5 and DRP1 was obviously increased in a dose-dependent manner, accompanied by a decrease in DRP1 serine 637 site phosphorylation in mitochondria.

Collectively, these results suggested that mPTP opening might involve iron overload-induced necroptosis in osteoblastic cells.

### 3.5. ROS Are Required for Iron Overload-Induced Necroptotic Cell Death in Osteoblastic Cells

**Figure 6:**
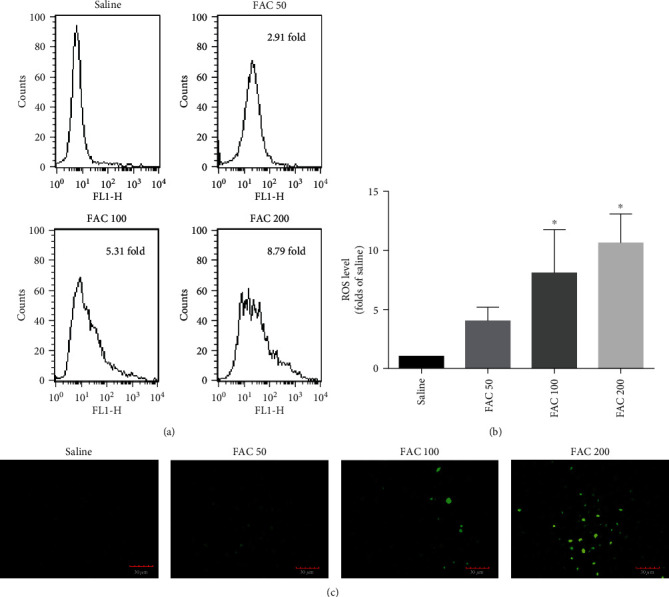
Iron overload induces the production of ROS in osteoblastic cells. (a) Representative data were analyzed using a flow cytometer after H2DCF-DA staining in osteoblasts after exposure to FAC (50-200 *μ*M) for 120 h. (b) Histogram statistical analysis illustrated the production of ROS in osteoblasts. Values are expressed as the means ± SD from three independent experiments (^∗^*p* < 0.05 vs. saline control, ANOVA test). (c) Representative fluorescence photomicrograph was obtained in situ H2DCF-DA staining by the fluorescence microscope. Scale bar = 30 *μ*m.

ROS caused by iron overload have long been considered the principal factor in regulating cell death [2]. To investigate whether ROS formation is required for iron overload-induced necroptosis in osteoblastic cells, we first measured the effect of iron overload on the intracellular ROS levels by flow cytometry. As shown in Figures 6(a) and 6(b), FAC increased the intracellular ROS levels in a dose-dependent manner. Furthermore, observation using fluorescence microscopy, the green fluorescence spots were dramatically increased in osteoblastic cells following FAC (50-200 *μ*M) treatment, indicating the generation of intracellular ROS induced by iron overload (Figure 6(c)).

**Figure 7:**
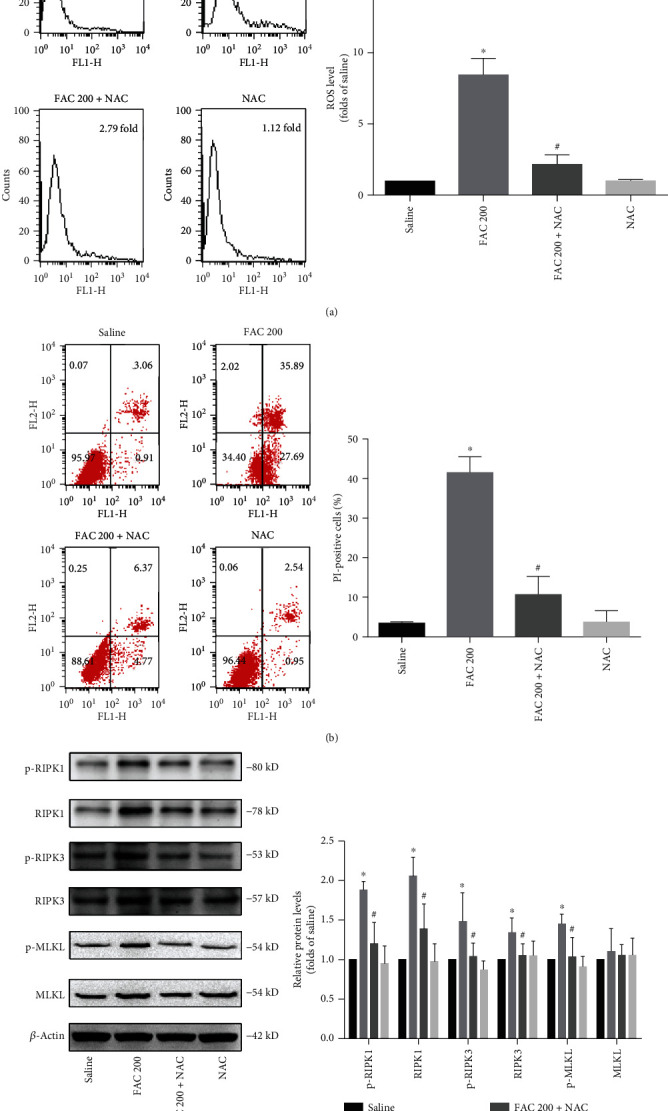
ROS are required for iron overload-induced necroptosis in osteoblastic cells. Osteoblasts were treated with 200 *μ*M FAC with or without NAC (1 mM) for 120 h. (a) Representative graphs were obtained from a flow cytometer after H2DCF-DA staining in osteoblasts. Histogram statistical analysis illustrated the production of ROS in osteoblasts. Data are expressed as the means ± SD from three independent experiments (^∗^*p* < 0.05 vs. saline control, ^#^*p* < 0.05 vs. FAC 200, ANOVA test). (b) Representative data were analyzed using a flow cytometer after Annexin-V/PI double staining in osteoblasts. Histogram analysis demonstrating the ratio of PI positive cells. Values are expressed as the means ± SD from three independent experiments (^∗^*p* < 0.05 vs. saline control, ^#^*p* < 0.05 vs. FAC 200, ANOVA test). (c) Representative western blots of the expression of phosphorylated RIPK1, RIPK3, MLKL, and corresponding total protein. (d) Histogram analysis showing the relative protein levels of phosphorylated RIPK1, RIPK3, MLKL, and corresponding total protein. Data are presented as the means ± SD from three independent experiments (^∗^*p* < 0.05 vs. saline control, ^#^*p* < 0.05 vs. FAC 200, ANOVA test).

Next, we explored whether ROS are indispensable for iron overload-induced necroptosis, using radical scavengers to inhibit the production of ROS. In our study, the antioxidant NAC significantly diminished the generation of ROS induced by iron overload in osteoblastic cells (Figure 7(a)). Notably, addition of NAC obviously reduced iron overload-induced necroptotic cell death of the osteoblastic cells (Figure 7(b)).

As the RIPK1-RIPK3-MLKL pathway represents an essential mechanism mediating necroptosis, we then analyzed whether ROS drive the necroptotic cell death through the RIPK1-RIPK3-MLKL pathway in osteoblastic cells. Intriguingly, we found that the increased phosphorylation of RIPK1, RIPK3, and MLKL induced by iron overload was abrogated by NAC. Besides, NAC also abolished the upregulated expression of the total protein of RIPK1 and RIPK3 (Figures 7(c) and 7(d)).

Together, this set of results confirms that ROS are required for iron overload-induced necroptotic cell death and might mediate the necroptosis through the RIPK1-RIPK3-MLKL pathway.

### 3.6. ROS Are Required for Iron Overload-Induced mPTP in Osteoblastic Cells

**Figure 8:**
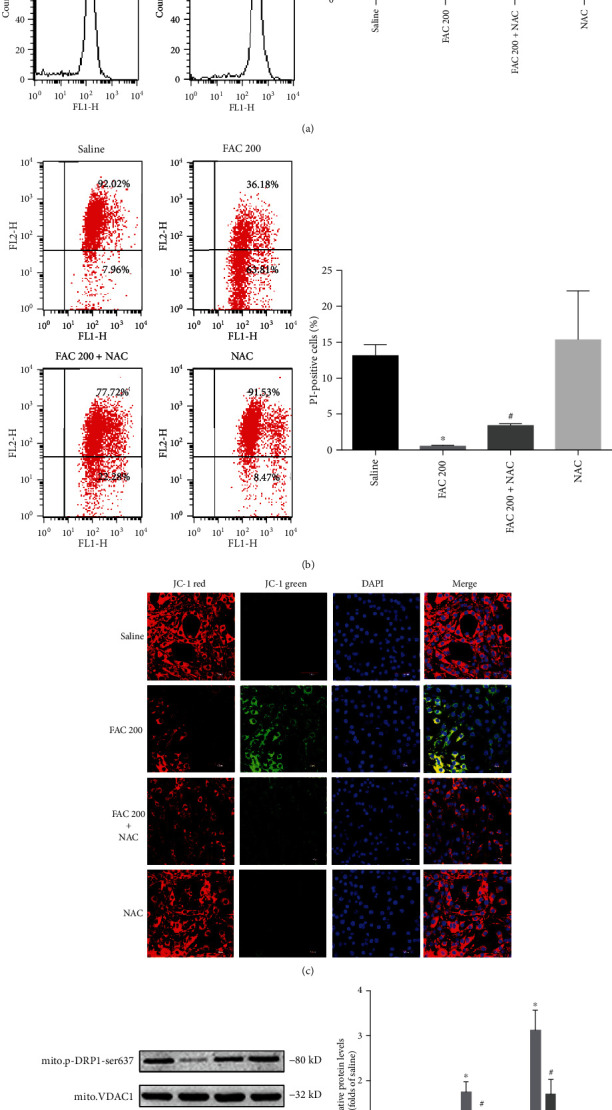
ROS are required for iron overload-induced mPTP opening in osteoblastic cells. The opening of mitochondrial permeability transition pore results in the loss of mitochondrial membrane potential. After exposure to FAC (200 *μ*M) with or without NAC (1 mM) for 120 h, the flow cytometry was used to detect the opening of mPTP and changes of MMP. (a) Representative dot plot of the flow cytometry results illustrated the increase mPTP opening. The quantitative mPTP in osteoblasts is shown by RFI. Data are expressed as the means ± SD from three independent experiments (^∗^*p* < 0.05 vs. saline control, ^#^*p* < 0.05 vs. FAC 200, ANOVA test). (b) Representative dot plot of the flow cytometry results illustrated the decrease MMP. The quantitative MMP in osteoblasts is shown by red/green ratio. Data are expressed as the means ± SD from three independent experiments (^∗^*p* < 0.05 vs. saline control, ^#^*p* < 0.05 vs. FAC 200, ANOVA test). (c) Representative fluorescence images were obtained in situ JC-1 staining by confocal microscopy. Scale bar = 50 *μ*m. (d) Representative western blots of the total protein expression of PGAM5 and DRP1 and mitochondrial protein expression of phosphorylated DRP1 (Ser637). (e) Histogram analysis showing the relative protein levels of PGAM5 and DRP1 and mitochondrial protein expression of phosphorylated DRP1 (Ser637). Data are presented as the means ± SD from three independent experiments (^∗^*p* < 0.05 vs. saline control, ^#^*p* < 0.05 vs. FAC 200, ANOVA test).

ROS has been confirmed as one of the principal factors triggering mPTP opening in multiple necrotic death pathways [33]. Once activating mPTP opening directly results in the loss of mitochondrial membrane potential, it eventually leads to necroptotic cell death [30]. To explore whether ROS generation is required for iron overload-induced mPTP, we used NAC to neutralize ROS stimulated by iron overload. As shown in Figure 8(a), FAC (200 *μ*M) promoted the opening of mPTP in osteoblastic cells, which was attenuated by NAC. Similarly, JC-1 staining revealed that NAC also could reverse the depolarization of MMP induced by iron overload in osteoblastic cells (Figures 8(b) and 8(c)). Furthermore, NAC inhibited the upregulation of PGAM5 and activation of DRP1 in mitochondria caused by iron overload (Figures 8(d) and 8(e)). Therefore, our results verified that ROS contributed to the mPTP opening under iron overload-induced necroptosis in osteoblastic cells.

### 3.7. RIPK1 And RIPK3 Contribute to Iron Overload-Induced ROS Generation in Osteoblastic Cells

**Figure 9:**
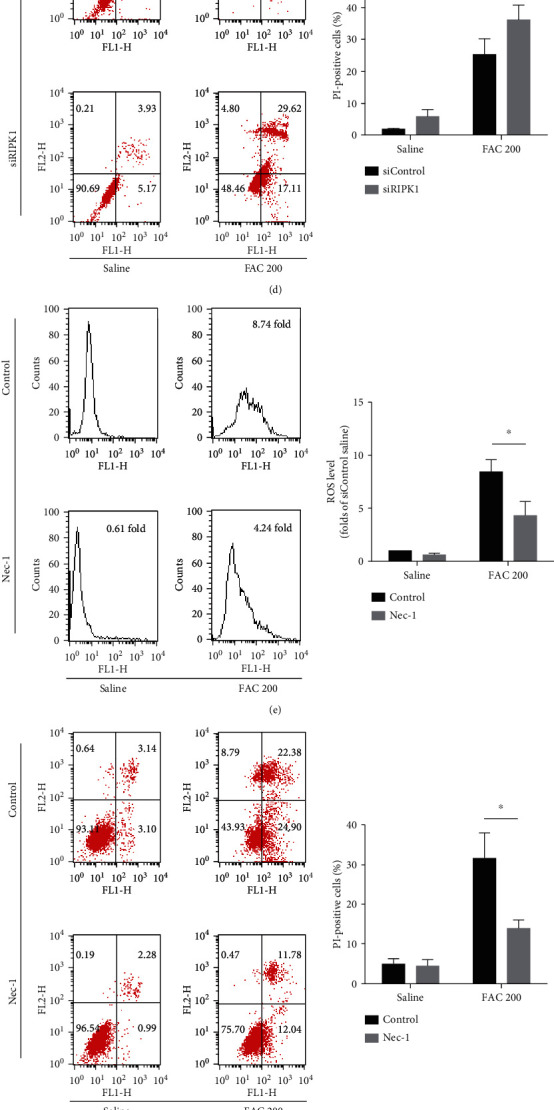
RIPK1 and RIPK3 contribute to iron overload-induced ROS. (a, b) The osteoblastic cells were transfected with siControl, siRIPK1, or siRIPK3 for 24 h; then, the expression of RIPK1 and RIPK3 was measured by western blot. Representative western blots of the expression of RIPK1 and RIPK3. Histogram analysis showing the relative protein levels of RIPK1 and RIPK3. Data are presented as the means ± SD of three independent experiments (^∗^*p* < 0.05 vs. siControl, Student's *t*-tests). (c, d) The osteoblastic cells were treated with FAC (200 *μ*M) for 120 h with siRIPK1 or siControl. Effect of siRIPK1 on ROS production by H2DCF-DA staining (c). Effect of siRIPK1 on iron overload-induced necrotic cell death by Annexin-V/PI double staining in the osteoblastic cells (d). (e, f) The osteoblastic cells were treated with FAC (200 *μ*M) for 120 h with or without Nec-1 (20 *μ*M). Effect of Nec-1 on ROS production by H2DCF-DA staining (e). Effect of Nec-1 on iron overload-induced necrotic cell death by Annexin-V/PI double staining in the osteoblastic cells (f). (g, h) The osteoblastic cells were treated with FAC (200 *μ*M) for 120 h with siRIPK3 or siControl. Effect of siRIPK3 on ROS production by H2DCF-DA staining (g). Effect of siRIPK3 on iron overload-induced necrotic cell death by Annexin-V/PI double staining in the osteoblastic cells (h). Data are expressed as the means ± SD from three independent experiments (^∗^*p* < 0.05 vs FAC 200, ANOVA test).

RIPK1 has been identified to contribute to the production of ROS and also play essential roles in the regulation of cell survival and death [34]. To analyze the roles of RIPK1 in iron overload-induced ROS production and cell death, RIPK1 was silenced with RIPK1siRNA or inhibited its kinase activity by Nec-1. First, we silenced RIPK1 as confirmed by western blot (Figure 9(a)). The generation of ROS in response to iron overload was significantly suppressed in the RIPK1-silenced and Nec-1-treated groups (Figures 9(c) and 9(e)). Interestingly, knockdown of RIPK1 significantly increased iron overload-induced necrotic cell death, which implied that RIPK1 deficiency in osteoblastic cells was highly sensitive to iron toxicity (Figure 9(d)). By comparison, Nec-1, described as the kinase activity inhibitor of RIPK1, significantly decreased iron overload-induced necrotic cell death (Figure 9(f)). Considering kinase-dependent and kinase-independent functions of RIPK1, our findings indicated that the kinase activity of RIPK1 might partly contribute to iron overload-induced ROS production and is required for iron overload-induced necroptosis in osteoblastic cells.

Next, we explored the role of RIPK3 in iron overload-triggered ROS generation, which has recently been considered a central regulator of necroptosis. To clarify the requirement of RIPK3 for iron overload-induced ROS generation, the osteoblastic cells were treated with RIPK3 siRNA. Knockdown efficacy of RIPK3 was confirmed by western blot analysis (Figure 9(b)). As shown in Figures 9(g) and 9(h), silencing of RIPK3 substantially attenuated iron overload-induced ROS production and also protected against iron overload-induced necrotic cell death. Meanwhile, inhibition of RIPK3 by GSK872 abrogated iron overload-stimulated necrotic cell death in the osteoblastic cells (Figures 4(c) and 4(d)). Based on our results, we conclude that RIPK3, at least in part, contributes to iron overload-induced ROS generation and is indispensable for iron overload-induced necroptosis in the osteoblastic cells.

## 4. Discussion

Osteoporosis is an age-related degenerative disorder that connected with a higher risk of fragility fractures. Iron gradually accumulates in the course of aging, and excess iron could accelerate bone loss in physically fit postmenopausal women and old men [35]. As numerous studies have confirmed iron overload as an independent risk contributor for the development of aging-associated osteoporosis, it is imperative to explore the precise iron toxicity in osteoblastic cells and elucidate the underlying mechanism. In the present study, we first demonstrated that iron overload could induce necroptosis of osteoblastic cells in vitro, which is mediated, at least in part, through the RIPK1/RIPK3/MLKL pathway. More interestingly, we revealed the critical regulation of ROS in iron overload-induced necroptosis. Notably, ROS induced by iron overload promote necroptosis via the formation positive feedback loop involving RIPK1/RIPK3.

Excessive iron can be toxic to many cell types, and it seems that the mechanism of iron toxicity is closely associated with cell death in the iron overload-related disorders [36, 37]. Historically, there are two mainly fundamental pathways of cell death: apoptosis and necrosis [38]. Apoptosis is marked by cytosolic shrinkage, nuclear condensation, apoptotic body formation, and activation of caspases [37]. The typical characteristics of necrosis, as opposed to apoptosis, are cellular swelling, plasma membrane disintegration, cellular contents release, and inflammation induction [14, 39]. Previous studies indicated that apoptosis mediated by the mitochondrial pathway was involved in iron overload-induced osteoblastic cell death [9]. However, our data suggested that the characteristics of osteoblastic cell death induced by iron overload seem to be closely related to necrosis, as proved by the higher PI-positive rate of cells and the typical necrotic morphological features obtained by TEM. Similar phenomena have also been found by previous work, which implied that necrosis might be the primary type of cell death for the osteoblastic cells in the iron overload-associated bone diseases [7, 40].

The exact mechanisms by which iron overload induced necrosis in osteoblastic cells have not been well established. Necrosis is traditionally considered to be an unregulated type of cell death, but growing evidence has shown that it is a type of programmed cell death carried out by multiple signaling transduction mechanisms [41]. Necroptosis, one type of programmed necrosis, was characterized by morphological changes of necrosis, which is critically dependent on the regulation of RIPK1, RIPK3, and MLKL. During activation of necroptosis, the phosphorylated RIPK1 recruits RIPK3 to form the RIPK1/RIPK3 necrosome complex. Then, RIPK3 recruits and phosphorylates MLKL [19]. Eventually, the phosphorylated MLKL undergoes oligomerization and translocates to the plasma membrane to execute necroptotic cell death [18, 29]. In this study, our data showed a dose-dependent increase in the total protein expression and phosphorylation of RIPK1 and RIPK3 in the osteoblastic cells after exposure to FAC. However, the protein expression of MLKL has no significant change in the osteoblastic cells after treatment with FAC. Considering RIPK3-dependent MLKL phosphorylation is the more critical step for necroptosis execution; we next detected the phosphorylation levels of MLKL in osteoblastic cells. In line with the changes of RIPK3 expression, we found that iron overload induced the increase of MLKL phosphorylation, indicating that phosphorylated MLKL is involved in the execution of necroptotic cell death. Furthermore, addition of Nec-1, GSK872, or NSA reduced iron overload-induced necrotic cell death in osteoblastic cells. Taken these results and previous research together, we could possibly conclude that iron overload promotes necroptosis in osteoblastic cells, at least in part, via the RIPK1/RIPK3/MLKL pathway.

RIPK1 and RIPK3 have been identified as two major executive molecules of necroptosis [11, 42]. Nevertheless, the exact mechanisms of RIPK1 and RIPK3 underlying osteoblastic necroptosis under iron overload condition are poorly understood. The molecular roles of RIPK1 in mediating necroptosis have been controversially discussed. On the one hand, numerous studies indicated that RIPK1 and its kinase activity are necessary for the initiation of necroptosis by various stimulators [43, 44]. On the other hand, RIPK1, through its kinase-independent scaffolding functions, maintains cellular homeostasis by inhibiting necroptosis [15, 45, 46]. In the current study, we showed that selective silencing of RIPK1 in osteoblastic cells sensitizes these cells to necroptosis triggered by iron overload. In contrast to genetic silencing of RIPK1, we indicated that Nec-1 protected against necroptosis induced by iron overload. Although these results seemed to be contradictory, we speculated this phenomenon might be related to kinase-dependent and kinase-independent functions of RIPK1 as discussed above. Furthermore, the exact molecular mechanisms of RIPK1 in mediating osteoblastic necroptosis will be investigated in the future studies. Currently, these results at least revealed that the kinase activity of RIPK1 was essential for iron overload-induced necroptosis in osteoblastic cells. Unlike RIPK1, the ability of RIPK3 in mediating necroptosis has been well established [47]. In our experiment, knockdown of RIPK3 significantly inhibited iron overload-induced necrotic cell death. Similarly, GSK872 also reduced iron overload-stimulated necrotic cell death in osteoblastic cells, which is consistent with previous studies. Based on these data together, we could possibly conclude that the kinase activity of RIPK1 and RIPK3 is indispensable for iron overload-induced necroptosis in osteoblastic cells.

In addition, mPTP opening has been also identified as an important downstream event for RIPK3-mediated necroptosis [29, 30]. PGAM5, a critical convergence effector of various necroptotic death pathways, could be recruited by phosphorylated RIPK3, then activates DRP1 dephosphorylation, and consequently promotes mPTP opening and necroptotic cell death [48–50]. More interestingly, our results revealed a dose-dependent increase in the levels of RIPK3 phosphorylation, upregulation of PGAM5 and DRP1, and dephosphorylation of DRP1 in mitochondria after treatment with FAC. Furthermore, we observed that the opening of mPTP was markedly increased and the PI-positive ratio of osteoblastic cells also concomitantly elevated. Therefore, we could be possibly concluded that the opening of mPTP is involved in necroptosis of osteoblastic cells in response to iron overload.

A key contribution of this study is the finding that ROS are essential for iron overload-induced necroptosis in osteoblastic cells. ROS have been considered as a critical driving force for necroptosis [19, 51]. Our previous studies have indicated that labile iron stimulated by iron overload contributed to the production of ROS in osteoblastic cells [9]. However, numerous evidence has suggested that the involvement of ROS in necroptosis is cell type-dependent [19]. It is therefore of great interest to explore whether ROS mediate iron overload-induced necroptosis in osteoblastic cells. Accordingly, we demonstrated that exposure with FAC triggered osteoblastic cells to produce ROS in a concentration-related manner. Furthermore, diminishing ROS by NAC strongly reduced iron overload-induced mPTP opening and necrotic cell death. In addition, posttranslational modifications involved by ROS play an essential role during necroptosis execution [19]. Furthermore, we detected the phosphorylation levels of RIPK1, RIPK3, and MLKL, three key upstream molecules of the necroptotic pathway. When NAC was applied, the phosphorylation levels of RIPK1 and RIPK3 dramatically decreased, indicating that ROS promote RIPK1 and RIPK3 phosphorylation in iron overload-induced necroptosis. Meanwhile, by detecting the necessity of key necroptotic molecules for ROS generation, we also found that RIPK1 and RIPK3 are indispensable for iron overload-induced ROS production, as genetic silencing of these molecules or inhibition of RIPK1 kinase activity by Nec-1 block ROS generation. Collectively, our results strongly suggest that ROS drive the phosphorylation of RIPK1 and RIPK3 and initiate a positive feedback loop involving RIPK1/RIPK3 in the iron overload-induced necroptosis process.

In our experiments, our data were highly reproducible. However, there are also some limitations that need to be considered. First, our study was conducted in vitro, and conclusions might not completely reflect the clinical conditions. In the subsequent studies, we will explore the exact mechanisms of iron toxicity in vivo. Second, because of technical limits for human osteoblastic cells, MC3T3-E1 cell line was used to investigate the cytotoxicity of iron [9, 52]. Third, our results only elaborate one aspect of iron toxicity pathway in osteoblastic cells. In fact, various pathways may contribute to the activation of iron overload-induced cell death. Therefore, in the subsequent studies, we need to further explore other pathways in iron overload-induced cell death of osteoblastic cells.

**Figure 10:**
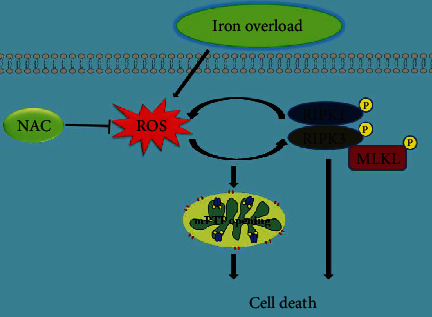
Schematic overview demonstrating that ROS-mediated necroptosis is involved in iron overload-caused osteoblastic cell death.

In conclusion, we firstly demonstrated that iron overload induced necroptosis of osteoblastic cells in vitro, which is mediated, at least in part, through the RIPK1/RIPK3/MLKL pathway. Moreover, we also revealed that ROS mediated iron overload-induced necroptosis via a positive feedback loop involving RIPK1/RIPK3. The possible mechanisms involved in iron overload-caused osteoblastic cell death are depicted in Figure 10. Importantly, our studies highlight the critical role of ROS in the regulation of iron overload-induced necroptosis in osteoblastic cells and provide a novel strategy to treat iron overload-associated bone disease.

## Figures and Tables

**Table 1:** 

Genes	The special sequences of siRNA
RIPK1 RIPK3	5′-AUGAUCUCCACGAUUAUCCdTdT-3′ 5′-GCAGUUGUAUAUGUUAAGGAGCGGUCGdTdT-3′

## Data Availability

The data used to support the findings of this study are available from the corresponding author upon request.

## Abbreviations

FAC: Ferric ammonium citrate
ROS: Reactive oxygen species
*α*-MEM: *α*-Minimum Essential Medium Eagle
mPTP: Mitochondrial permeability transition pore
NAC: N-Acetylcysteine
MMP: Mitochondrial membrane potential
RIPK1: Receptor-interacting protein kinase 1
MLKL: Mixed lineage kinase domain-like
RIPK3: Receptor-interacting protein kinase 3
FBS: Fetal bovine serum
Nec-1: Necrostatin-1
NSA: Necrosulfonamide
TEM: Transmission electron microscopy
siRNA: Small interfering RNA
JC-1: 5′,6,6′-Tetrachloro-1,1′,3,3′-tetraethyl-benzimidazolycarbocyanine iodide
CCK-8: Cell counting kit-8.

## Supplementary Materials

Supplementary 1 Supplementary Figure 1: the protective effects of Nec-1 against iron overload-induced cytotoxity in osteoblastic cells. After subjecting to FAC (200 *μ*M) with or without Nec-1 (5-160 *μ*M) for 120 h, the viability of osteoblastic cells was detected by CCK-8 assays. Values are expressed as the means ± SD from three independent experiments (^∗^*p* < 0.05 vs. saline control; ^#^*p* < 0.05 vs. FAC 200).

Supplementary 2 Supplementary Figure 2: the protective effects of GSK872 against iron overload-induced cytotoxity in osteoblastic cells. After subjecting to FAC (200 *μ*M) with or without GSK872 (1-32 *μ*M) for 120 h, the viability of osteoblastic cells was detected by CCK-8 assays. Values are expressed as the means ± SD from three independent experiments (^∗^*p* < 0.05 vs. saline control; ^#^*p* < 0.05 vs. FAC 200).

Supplementary 3 Supplementary Figure 3: the protective effects of NSA against iron overload-induced cytotoxity in osteoblastic cells. After subjecting to FAC (200 *μ*M) with or without NSA (1-32 *μ*M) for 120 h, the viability of osteoblastic cells was detected by CCK-8 assays. Values are expressed as the means ± SD from three independent experiments (^∗^*p* < 0.05 vs. saline control; ^#^*p* < 0.05 vs. FAC 200) (Supplementary Materials).
