# Supplementary figures and images for: ROS-Mediated Necroptosis Is Involved in Iron Overload-Induced Osteoblastic Cell Death
Source: Oxid Med Cell Longev. 2020 Oct 16;2020:1295382. doi: 10.1155/2020/1295382 (PMC7586162; doi:10.1155/2020/1295382)

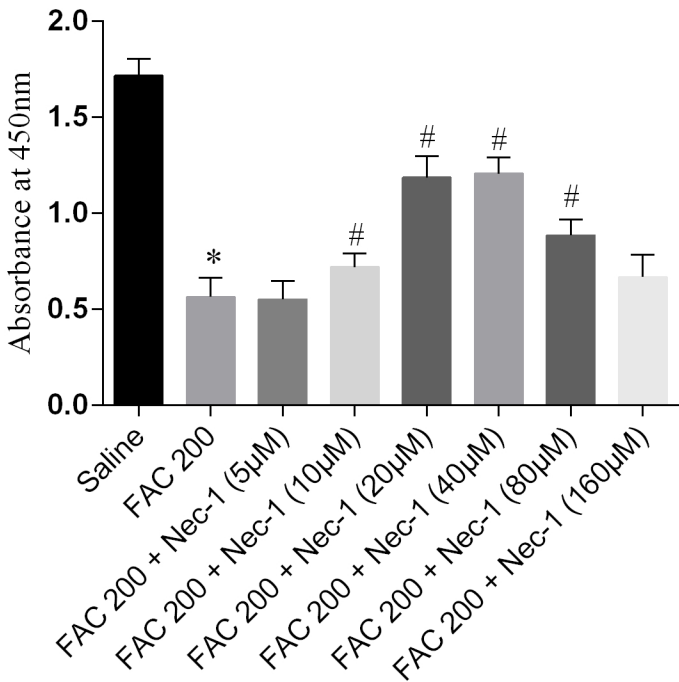

Supplement: Supplementary 1 — Supplementary Figure 1: the protective effects of Nec-1 against iron overload-induced cytotoxity in osteoblastic cells. After subjecting to FAC (200 μM) with or without Nec-1 (5-160 μM) for 120 h, the viability of osteoblastic cells was detected by CCK-8 assays. Values are expressed as the means ± SD from three independent experiments (∗p < 0.05 vs. saline control; #p < 0.05 vs. FAC 200). [file 1295382.f1.pdf]

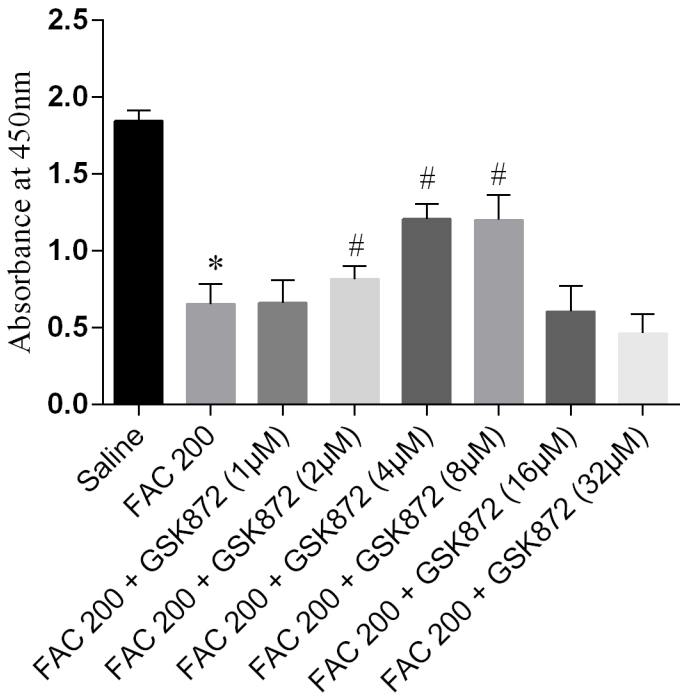

Supplement: Supplementary 2 — Supplementary Figure 2: the protective effects of GSK872 against iron overload-induced cytotoxity in osteoblastic cells. After subjecting to FAC (200 μM) with or without GSK872 (1-32 μM) for 120 h, the viability of osteoblastic cells was detected by CCK-8 assays. Values are expressed as the means ± SD from three independent experiments (∗p < 0.05 vs. saline control; #p < 0.05 vs. FAC 200). [file 1295382.f2.pdf]

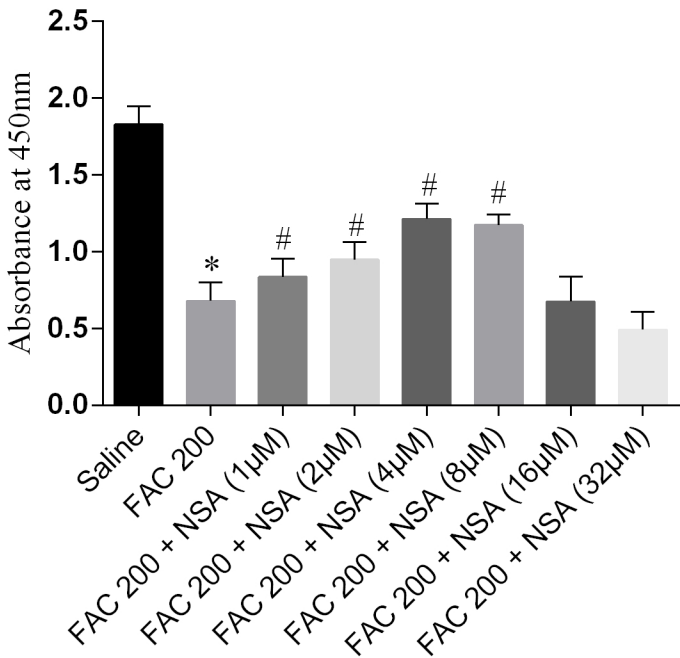

Supplement: Supplementary 3 — Supplementary Figure 3: the protective effects of NSA against iron overload-induced cytotoxity in osteoblastic cells. After subjecting to FAC (200 μM) with or without NSA (1-32 μM) for 120 h, the viability of osteoblastic cells was detected by CCK-8 assays. Values are expressed as the means ± SD from three independent experiments (∗p < 0.05 vs. saline control; #p < 0.05 vs. FAC 200) (Supplementary Materials). [file 1295382.f3.pdf]
